# Comparison of iSeq and MiSeq as the two platforms for 16S rRNA sequencing in the study of the gut of rat microbiome

**DOI:** 10.1007/s00253-022-12251-z

**Published:** 2022-11-02

**Authors:** Dominika Salamon, Barbara Zapała, Agnieszka Krawczyk, Agnieszka Potasiewicz, Agnieszka Nikiforuk, Anastazja Stój, Tomasz Gosiewski

**Affiliations:** 1 Department of Molecular Medical Microbiology, Jagiellonian University Medical College, Krakow, Poland; 2 Department of Clinical Biochemistry, Jagiellonian University Medical College, Krakow, Poland; 3 Department of Behavioural Neuroscience and Drug Development, Polish Academy of Sciences, Maj Institute of Pharmacology, Krakow, Poland; 4 Department of Hematology Diagnostics and Genetics, University Hospital, Krakow, Poland

**Keywords:** MiSeq, iSeq, 16S rRNA sequencing, Gut microbiome, Abundance, Biodiversity, NGS

## Abstract

**Abstract:**

Amplicon-based next-generation sequencing (NGS) of the 16S ribosomal RNA (16S) regions is a culture-free method used to identify and analyze *Procaryota* occurring within a given sample. The prokaryotic 16S rRNA gene contains conserved regions and nine variable regions (V1-V9) frequently used for phylogenetic classification of genus or species in diverse microbial populations. This work compares the accuracy and efficacy of two platforms, iSeq and MiSeq from Illumina, used in sequencing 16S rRNA. The most important similarities and differences of 16S microbiome sequencing in 20 fecal rat samples were described. Genetic libraries were prepared according to 16S Metagenomic Sequencing Library Preparation (Illumina) for the V3 and V4 regions of the 16S. The species richness obtained using iSeq technology was lower compared to MiSeq. At the second taxonomy level (L2), the abundance of taxa was comparable for both platforms. At the L7, the taxa abundance was significantly different, and the number of taxa was higher for the MiSeq. The alpha diversity was lower for iSeq than for MiSeq, starting from the order to the species level. The beta diversity estimation revealed statistically significant differences in microbiota diversity starting from the class level to the species level in samples sequenced on two investigated platforms. This work disclosed that the iSeq platform could be used to evaluate the bacterial profile of the samples to characterize the overall profile. The MiSeq System seems to be better for a detailed analysis of the differences in the microbiota composition.

**Key points:**

*• iSeq platform allows to shorten the sequencing time three times compared to the MiSeq.*

*• iSeq can only be used for an initial and quick microbiome assessment.*

*• MiSeq is better for a detailed analysis of the differences in the microbiota composition.*

**Supplementary information:**

The online version contains supplementary material available at 10.1007/s00253-022-12251-z.

## Introduction

Due to the increasing interest in the microbiome and its relationship within the different ecosystems, it is crucial to establish the appropriate method that will allow for a precise assessment of the microorganisms’ composition in various ecological niches. Research on the microbiome includes its relationship with the natural environment (Cabello-Yeves et al. 2021) and in the context of animal breeding (Vasco et al. 2021) and agricultural crops (Zhang et al. 2021). As it was described in many reports using both animal and human models, the gut microbiome is the most complex, often studied and correlated with many diseases such as diabetes (Salamon et al. 2018, 2021), obesity (Sroka-Oleksiak et al. 2020), Crohn’s disease (Kowalska-Duplaga et al. 2019), and skin diseases or schizophrenia (Szeligowski et al. 2020). It is known that many microorganisms are difficult to culture, e.g., in the gut (Lau et al. 2016) or hydrothermal sources (Bellec et al. 2020), so they are hard to detect in different samples. Therefore, the optimal method in such studies seems to be next-generation sequencing (NGS), high-throughput sequencing. The most common taxa identification is based on the amplicon-based NGS of the 16S ribosomal RNA (rRNA) gene. The prokaryotic 16S rRNA gene is approximately 1500 bp long and has conserved and hypervariable nine regions (V1-V9), which are used for phylogenetic classification of genera and in diverse microbial populations (Gao et al. 2021; Nakao et al. 2021). Such metagenomic studies require specific infrastructure, trained personnel, appropriate sequencing platforms, and bioinformatics pipelines (Allali et al. 2017; Gao et al. 2021). Since 2011, many laboratories have used the Illumina NGS technology, especially the MiSeq system (San Diego, CA, USA), which is the most commonly used, enabling the evaluation of relatively short paired-end sequencing reads with high accuracy (https://www.illumina.com/content/dam/illumina/gcs/assembled-assets/marketing-literature/miseq-system-data-sheet-m-gl-00006/miseq-data-sheet-m-gl-00006.pdf; accessed on September 25, 2022). In 2018, the company introduced the new iSeq 100 system, which can be used for high-throughput sequencing of the 16S rRNA gene. The low cost and small size of the iSeq machine create a new opportunity for many laboratories, even being an alternative to the MiSeq platform (https://www.illumina.com/content/dam/illumina-marketing/documents/products/datasheets/iseq100-sequencing-system-spec-sheet-770-2017-020.pdf; accessed on September 25, 2022). Based on the data provided by Illumina, the most important characteristics of the two above-mentioned platforms are presented in Table 1.

**Table 1:** Key features of the 2 selected NGS (Illumina, San Diego, CA, USA) platforms in the field of 16S sequencing [based on references: (https://jornades.uab.cat/workshopmrama/sites/jornades.uab.cat.workshopmrama/files/16s_sequencing.pdf; accessed on September 25, 2022; Hu et al. 2021)]

Platform	iSeq 100 system	MiSeq system
Dimensions in inches HxWxD (in cm)	16.8 × 12 × 13 (42.5 × 30.5 × 33)	20.6 × 27 × 22.2 (52.3 × 68.6 × 56.5)
Price in $	~ 19,900	~ 99,000
Samples/run	1–48	1–96
Cost per run (in $)	~ 495 (pricing based on iSeq 8-pack reagent kit)	~ 325–1705
Run time	9.5–19 h	~ 55 h
Maximum output	1.2 Gb	15 Gb
Maximum reads per run	4 million	25 million
Maximum read length	2 × 150 bp	2 × 300 bp
Quality scores	> 80% bases higher than Q30	> 70% bases higher than Q30
Reads passing filter (PF)/run	4 million	44–50 million
Optimal raw cluster density (k/mm^2^)	System utilizes patterned flow cells which result in a fixed cluster density	1200–1400

This study aimed to compare the accuracy and efficacy of two selected iSeq and MiSeq, Illumina machines in sequencing 16S rRNA (16S) in the same samples of the rat schizophrenia model. Furthermore, using the two different iSeq and MiSeq, Illumina technologies for sequencing 16S rRNA in the same schizophrenia rats’ feces samples, the potential differences in the most important characteristics (in the field of richness and evenness (alpha diversity) and differences in microbial structure (beta diversity) were compared.

## Materials and methods

### Samples and DNA isolation

We used 20 fecal samples taken from rats while running another project (“The effects of ligands of the alpha7 nicotinic acetylcholine receptors on complex cognitive processes and social behavior in the neurodevelopmental schizophrenia-like model”, project grant no 014/15/N/NZ7/02978 supported by National Science Centre, approval of the 2nd Local Institutional Animal Care and Use Committee in Krakow, no 1198/2015) on gut microbiota in a rat model of schizophrenia. Samples were collected in polypropylene tubes (FL Medical, Padova, Italy) and immediately frozen at − 80 °C pending further analysis, for no longer than 2 months. Bacterial DNA was isolated according to our previous works using mechanical, chemical, and enzymatic lysis of microbial cells and commercial Genomic Mini AX Stool Spin kit (A&A Biotechnology, Gdansk, Poland) (Gosiewski et al. 2014; Sroka-Oleksiak et al. 2020; Krawczyk et al. 2021). The concentration and purity of DNA isolates were determined spectrophotometrically for the A260 nm and A260nm/280 nm ratio using NanoDrop (Thermofisher, Waltham, MA USA).

### Library preparation

The 16S libraries were amplified by PCR. Primers targeting the 16S rRNA gene hypervariable V3 and V4 regions with the overhang adapter sequences (Klindworth et al. 2013). The composition of the reaction mixture and the thermal amplification profiles were presented in Supplemental Table S1.

Further, the PCR product (550 bp) was verified using agarose gel electrophoresis.

Libraries were prepared following the protocol for Preparing 16S Ribosomal RNA Gene Amplicons for Illumina MiSeq System (Part#15044223Rev.B) (http://support.illumina.com/content/dam/illumina-support/documents/documentation/chemistry_documentation/16s/16s-metagenomic-library-prep-guide-15044223-b.pdf; accessed on September 25, 2022). First, according to the manufacturer’s recommendations, the PCR-based amplification was performed using KAPA HiFi HotStart ReadyMix (Roche, Basel, Switzerland). Then amplicons were pooled in equimolar concentrations of 10 pM for MiSeq and 55 pM for iSeq sequencing. Finally, both pooled libraries were diluted with 10 mM Tris pH 8.5 to the concentration of 4 nM. The differences and the details in library preparations are described in Table 2.

**Table 2:** The differences and the details in library preparations in final stage for two sequencers

Sequencer	MiSeq	iSeq
Library denaturing	With NaOH	Without NaOH
Final concentration of denatured library	10 pM	55 pM
Mix of amplicon library and PhiX control final volume	600 µl	20 µl
Spike-in PhiX control DNA volume	30%	10%
Kind of cartridge	Open model	Close model (after sequencing, the cassette is disposed of, therefore apparatus rinsing is not required)

### Next-generation sequencing

The libraries containing 20 pooled indexed samples with 30% spike-in PhiX (Illumina San Diego, CA, USA) control DNA were loaded into the libraries for MiSeq and 10% onto libraries for iSeq. Sequencing was performed using MiSeq Reagent Kit v3 (600-cycle) and iSeq 100 i1 Reagent v2 (300-cycle) respectively.

### Bioinformatics and statistical analysis

The data generated from MiSeq and iSeq as raw reads in FASTQ formats were filtered using the Illumina 16S Metagenomics workflow. Then the high-quality sequences were clustered, and the operational taxonomic units (OTUs) with 99.9% identity were prepared based on the Greengenes Database and the algorithm with the high-performance implementation of the Ribosomal Database Project (RDP) classifier, described by Wang et al. (2007). The data filtering was performed at the beginning of the downstream statistical analysis. The features with identical or zero values and the artifacts across all samples were excluded from further analysis. Next, the data with low quality and uninformative features were removed. A low count filter was adjusted with a prevalence of at least 20%, and features with minimal counts (< 4) were excepted from the analysis to exclude sequencing errors. In the next step of downstream analysis, data were normalized, including the variability in sampling depth and the sparsity of the data. After the normalization, a meaningful biological comparison was performed, and species richness was compared. All samples were rarefied to sequencing depth based on the sample having the lowest sequencing depth to normalize the data. Then LEfSe (Segata et al. 2011) and Microbiome Analyst platform (Chong et al. 2020) was applied in the next clustering steps and further statistical analysis. The most characteristic features were determined using the linear discriminant analysis (LDA) effect size with LEfSe (Segata et al. 2011). Using LEfSe, we described the highest statistical and biological differences between samples sequenced with MiSeq and iSeq. The discovered statistical significance and biological relevance were described based on the normalized relative abundance matrix, the Kruskal–Wallis rank-sum test, the significant alpha at 0.05, and the effect size threshold of 2. The hierarchical structure of taxonomic classifications was characterized using the median abundance and the non-parametric Wilcoxon rank-sum test to show taxonomic differences between microbial communities and abundance profiles of two experimental groups (Foster et al. 2017). Alpha and beta diversity were calculated using QIIME 2.0 software with Python scripts (Schloss et al. 2009). Alpha diversity was calculated based on the sequence similarity at the 97% level. The richness was calculated as the amount of unique OTUs found in each sample and presented as observed OTUs, and the count of unobserved species based on low-abundance OTUs was visualized as ACE and Chao1 indices. Shannon, Simpson, and Fisher estimators were calculated to measure both the richness and evenness within individual samples and in the experimental groups of samples. Beta diversity was determined as the distance and dissimilarities in-between microbial communities based on Jaccard, Bray–Curtis, and Jensen-Shannon Divergence indices calculated by QIIME. The distances were visualized by principal coordinate analysis (PCoA). Differences based on beta diversity of the whole microbiome structure among groups were calculated using a permutational multivariate analysis of variance (PERMANOVA). The similarity and dissimilarity were measured based on Pearson’s Correlation Coefficient. The dissimilarities, showing the distances between samples, were calculated based on the Jensen-Shannon Divergence beta diversity metric.

The sequence data (fastq files) has been deposited in The Jagiellonian University Repository—online access: https://ruj.uj.edu.pl/xmlui/handle/item/298521.

## Results

DNA samples with a purity ≥ 1.7 were selected for further testing and all DNA samples tested fell within this range. The twenty fecal samples of rats were sequences on iSeq, and then the same samples were sequenced on the MiSeq machine.

Table 3 demonstrates the detailed run characteristics for both iSeq and MiSeq sequencing. We showed the differences, especially in maximum read counts per sample and the number of identified species higher for MiSeq (Table 3).

**Table 3:** The detailed summary of reading counts calculated for each sample and detailed run characteristics for two sequencers

Platform	iSeq 100 system	MiSeq system
Run time	11 h	50 h
% total bases *Q* ≥ 30	> 92.24% bases higher than Q30	> 84.24% bases higher than Q30
% bases *Q* ≥ 30 read 1	93.24%	88.37%
% bases *Q* ≥ 30 Read 2	78.62%	57.65%
% passing filter (PF)	74%	92%
Density raw (k/mm^2^)	327	780
Cluster count raw (k)	482	523
Density PF (k/mm^2^)	242	712
Cluster count PF (k)	356	480
Error rate	0.45%	2.6%
Total reads	16,565,235	19,611,220
Average reads per sample	52,300	54,029
Median	288	309
Minimum	14,338	507
Maximum	85,005	197,214
Mean of number reads PF	115	83
Mean of % reads PF classified to genus	93	92
Number of species identified	7026	9184
Total OTUs	1,046,010	1,080,598
At L6	1,508,570	2,135,705
At L7	1,605,237	2,247,416

**Fig. 1:**
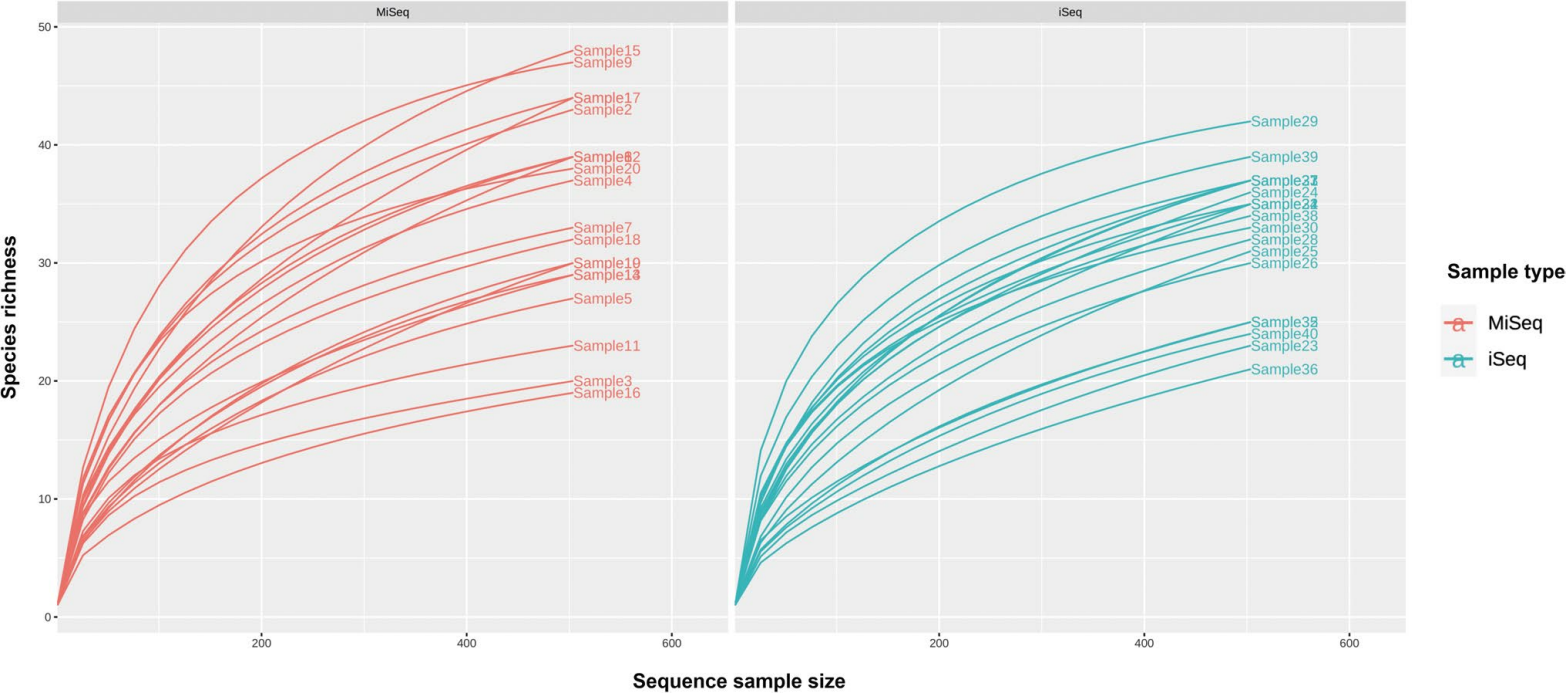
The rarefaction curves for each group in two separate plots show the species richness in samples sequenced in iSeq and MiSeq machines. First, the data and raw sequencing reads were mapped against the complete gene catalog to assess sampling depth and sparsity variability. Then the gene recovery for different numbers of reads was calculated and plotted in the form of a rarefaction curve. The rarefaction curves created for samples sequenced in two different machines show the increasing numbers of raw sequencing reads and much more variation in discovered gene content in samples analyzed by MiSeq than variation attributed to samples sequenced in iSeq. The rarefaction curves of iSeq results reach a plateau earlier than those of MiSeq, which means that only a few new sequences are detected with increasing sequencing depth in iSeq. There was a difference in unequal sequencing depths in MiSeq and iSeq, influencing the richness between microbial communities measured in the samples. Rarefaction curves were obtained based on the R statistical programming language run on a high-performance computing cluster

The statistically significant differences were found in species richness, with higher numbers of species detected using the MiSeq compared to iSeq (*p* < 0.01). Moreover, the most OTUs detected on iSeq platform represented the same species. The differences in species richness are shown in Fig. 1, demonstrating rarefaction curves for both iSeq and MiSeq sequencing results.

**Fig. 2:**
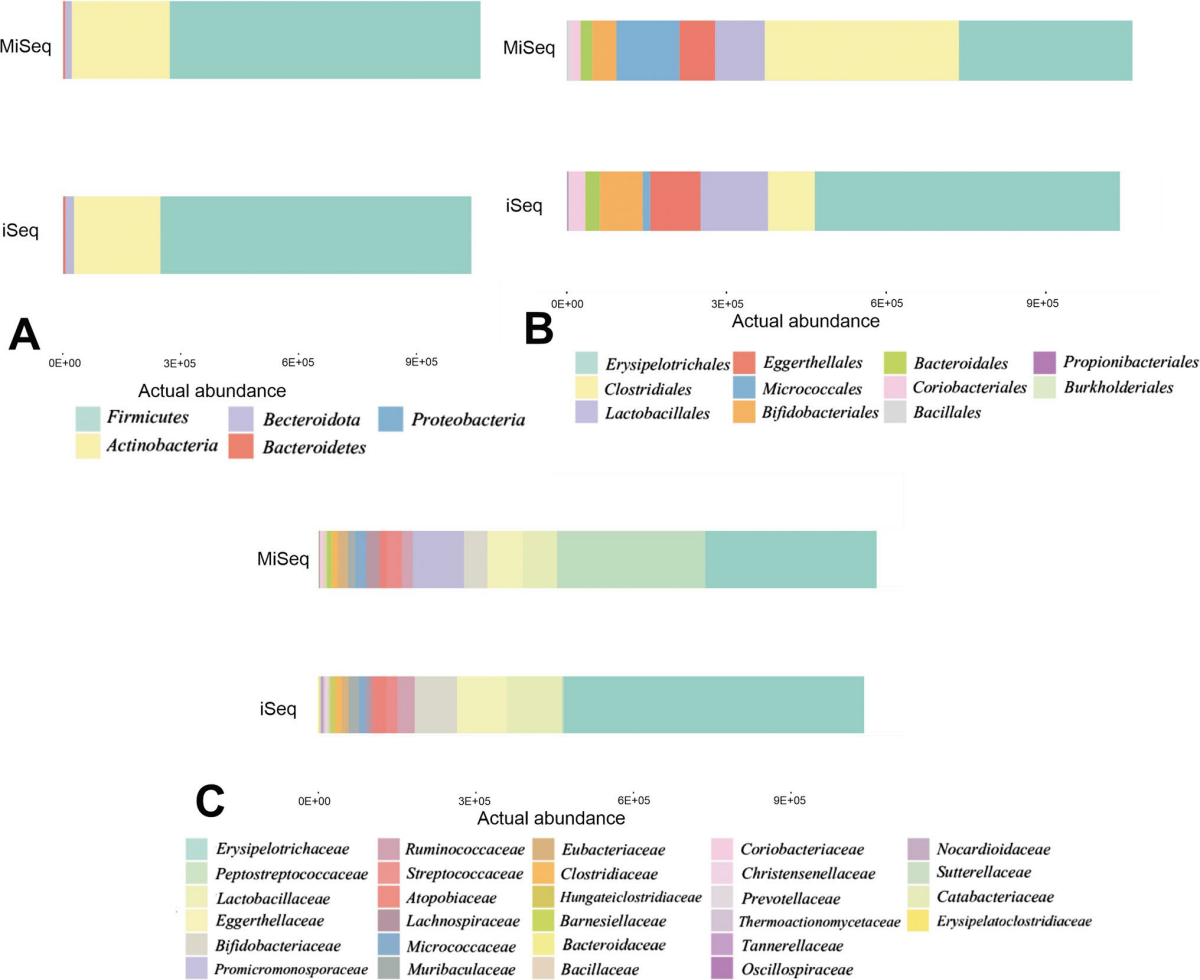
Different taxonomic levels differences in the actual abundance (the taxa were merged based on the sum of their counts across all samples categorized to the experimental groups) of samples sequenced on MiSeq and iSeq at different taxonomic levels, **A** phylum, **B** class, **C** genus

The OTU data were summarized and compared based on their abundance at different taxonomic levels (Fig. 2). The significant differences in detected OTUs were noticed at all taxonomic levels except L2 (Fig. 2A). At the class level–L3 (Fig. 2B), the results for the MiSeq showed a higher abundance of *Clostridia* compared to iSeq (*p* < 0.001), and from the iSeq platform a higher abundance of *Erysipelotrichia* than for MiSeq (*p* < 0.001). At L4 (order level), the abundance of *Bifidobacterales* (80,542 reads vs 44,994 reads, *p* = 0.04), *Erysipelotrichales* (5,73,282 reads vs 326,310 reads, *p* < 0.001), and *Propionibacteriales* (1848 reads vs 157 reads, *p* < 0.001) was higher for the iSeq than from MiSeq respectively. The abundance of *Clostridiales* was higher for MiSeq (364,839 reads) than iSeq (87,868 reads, *p* < 0.001). The statistically significant differences in the OTUs abundance at the levels family (L5), genus (L6) (Fig. 2C), and species (L7) were detailed in Supplemental Table S2.

**Fig. 3:**
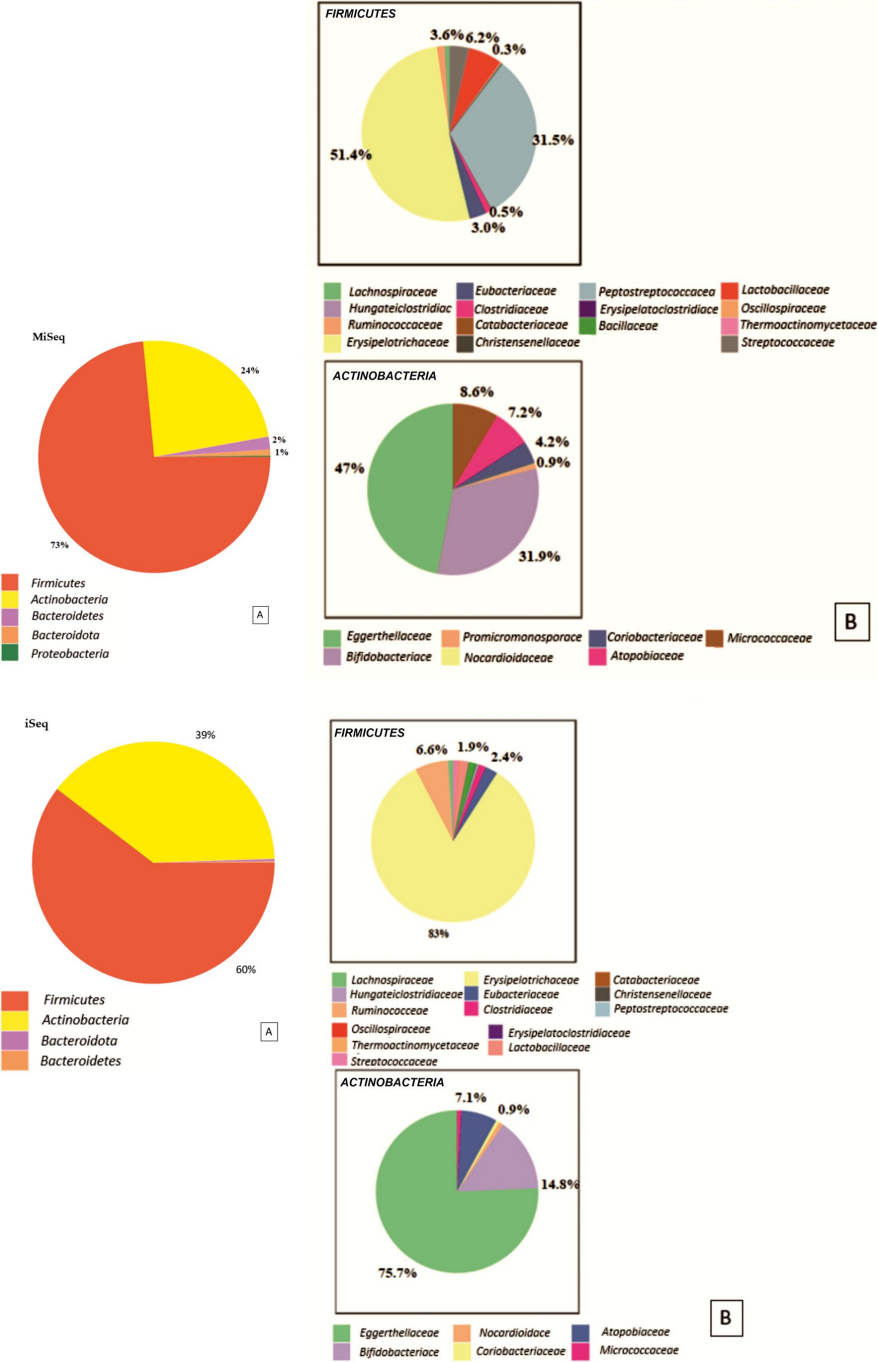
The pie charts show the abundance profiles of one randomly selected sample (ME9okMiSeq) at two taxonomic levels: phylum (**A**) and family (**B**) of the two selected phyla: *Firmicutes* and *Actinobacteria* sequenced with MiSeq and iSeq

The statistically significant differences in the abundance profiles were detected when comparing the results of the characteristics of the taxonomic profile of one randomly selected sample (ME9dkMiSeq/ME9dkiSeq) obtained from both tested sequencers. As visualized in Fig. 3A, B, the results from MiSeq were characterized by a higher abundance of *Firmicutes* phylum (73%) (Fig. 3A, B) than those from iSeq (60%) (Fig. 3A, B). In contrast, the *Actinobacteria* phylum was less abundant (24%) in the sample sequenced in MiSeq (Fig. 3A, B) compared to the iSeq (39%) (Fig. 3A, B).

**Fig. 4:**
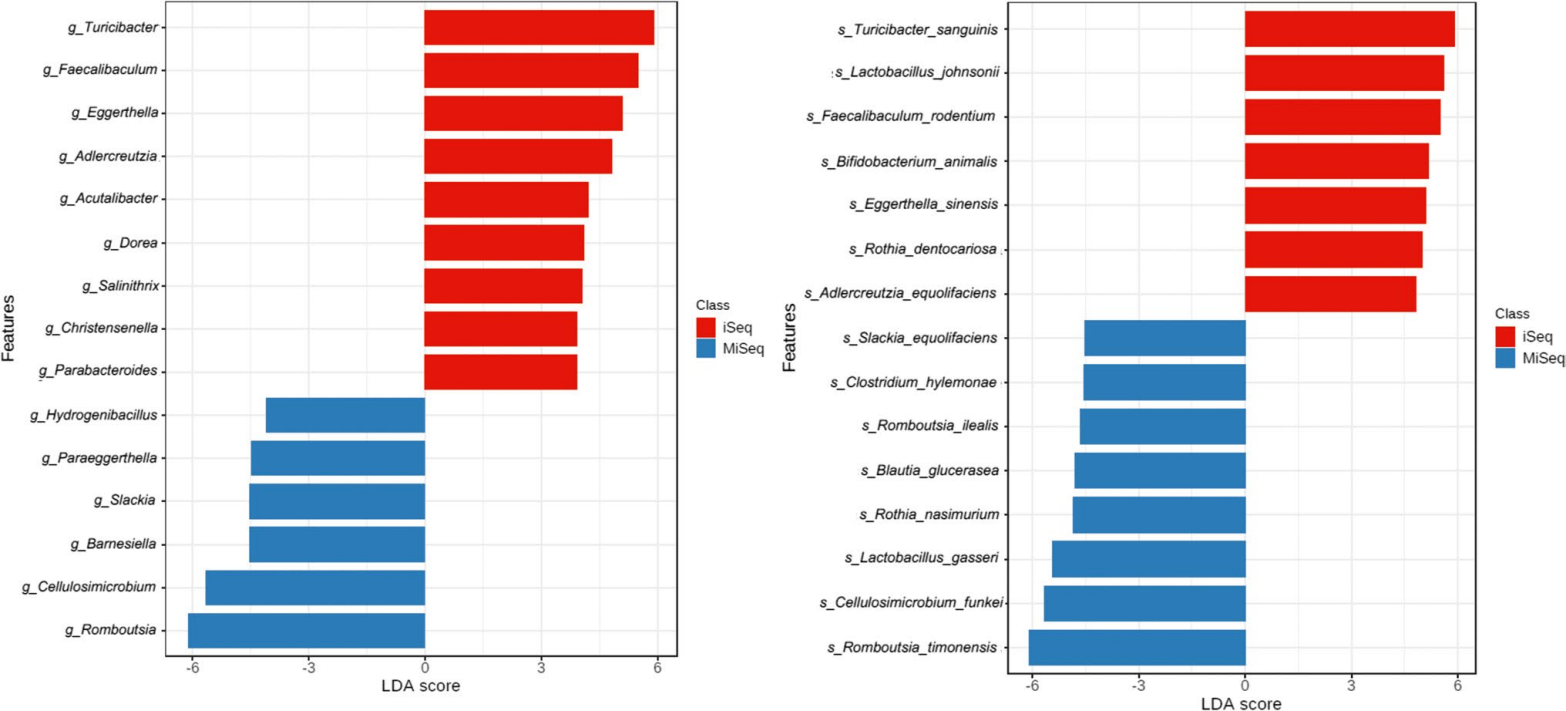
The statistically significant biomarkers discovery results with regard to genus – L6 (**A**), and species – L7 level (**B**) for the same samples sequenced using iSeq and MiSeq systems demonstrated by colored bar plots. The graphical output of the LDA score demonstrates both the negative and positive values, with the cutoff of 2.0 determining the most significant features

The linear discriminant analysis (LDA) revealed statistical significance in the biomarkers discovery. Furthermore, using the Kruskal–Wallis rank sum test, the features with significant differential abundance were identified, showing the discrepancies in the interpretation of metagenomic data obtained for the same samples in iSeq or MiSeq systems. These significant differential abundances were detected with regard to the following taxonomic levels: class (*Clostridia*, *p* < 0.000; *Erysipelotrichia*, *p* < 0.000), order (*Clostridiales*, *p* < 0.000; *Propionibacteriales*, *p* < 0.000; *Erysipelotrichales*, *p* < 0.000; *Bifidobacteriales*, *p* < 0.004), family (*Promicromonosporaceae*, *p* < 0.000; *Bacillaceae*, *p* < 0.000; *Peptostreptococcaceae*, p < 0.000; *Erysipelatoclostridiaceae*, *p* < 0.000; *Thermoactinomycetaceae*, *p* < 0.000; *Nocardioidaceae*, *p* < 0.000; *Barnesiellaceae*, *p* < 0.000; *Erysipelotrichaceae*, *p* < 0.002; *Christensenellaceae*, *p* < 0.005; *Tannerellaceae*, *p* < 0.024; *Bacteroidaceae*, *p* < 0.039), genus (*p* < 0.05), and species (*p* < 0.05). For details, see Supplemental Table S3 and S4. The 15 genus and species with the highest significant differential abundance were selected to be shown in Fig. 4.

The alpha diversity profiling also showed the statistical significance in the diversity of the same samples sequenced in iSeq or MiSeq sequencers. The significance testing of alpha diversity within the same samples sequenced in iSeq and MiSeq machines showed statistically significant differences starting from the order level (Table 4). Moreover, the alpha diversity metrics for results obtained from MiSeq were higher when compared to iSeq, excluding phylum and class levels (Supplemental Figs. S1 to S5). The most common metrics which were used to calculate the diversity within the same samples sequenced in iSeq and MiSeq are shown in Table 4.

**Table 4:** The *p*-value of alpha diversity measurements using the most common metrics in the same samples sequenced in iSeq and MiSeq machines

Taxonomic level	Alfa diversity metrics *p*-values (MiSeq vs iSeq)					
	Observed	ACE	Chao1	Shannon	Simpson	Fisher
L2 (phylum)	0.643	0.568	0.643	0.325	0.277	0.195
L3 (class)	0.643	0.568	0.643	0.325	0.277	0.195
L4 (order)	0.003*	0.775	0.014*	0.273	0.033*	0.959
L5 (family)	0.032*	0.077	0.051	0.052	0.004*	0.015*
L6 (genus)	0.09	< 0.001*	0.016*	0.514	0.118	0.024*
L7 (species)	< 0.001*	< 0.001*	< 0.001*	0.341	0.088	< 0.001*

^*^Statistically significant value.

The comparison of microbial communities (in-between) based on their compositions revealed statistically significant. Table 5 demonstrated the beta diversity of the same samples sequenced in both iSeq and MiSeq machines. Statistically significant differences in microbiota composition of the same samples sequenced in two different sequencers were detectable starting from the order level (Table 5, Supplemental Figs. S6 to S11).

**Table 5:** The *F*-values, *R*-squared values, and *p*-values of beta diversity significance testing in the same samples sequenced in iSeq and MiSeq machines

Taxonomic level	Beta diversity indices (MiSeq vs iSeq)								
	Bray–Curtis			Jensen-Shannon			Jaccard		
	*F*-value	*R*-squared	*p*-value	*F*-value	*R*-squared	*p*-value	*F*-value	*R*-squared	*p*-value
L2 (phylum)	0.11248	0.0029513	0.82	− 2.322	− 0.065082	0.991	0.76578	0.019754	0.447
L3 (class)	0.11248	0.0029513	0.82	− 2.322	− 0.065082	0.991	0.76578	0.019754	0.447
L4 (order)	14.214	0.27222	0.001*	15.589	0.2909	0.001*	12.605	0.24908	0.001*
L5 (family)	14.115	0.27084	0.001*	16.27	0.29979	0.001*	12.182	0.24275	0.001*
L6 (genus)	15.881	0.29474	0.001*	24.182	0.38889	0.001*	13.128	0.25676	0.001*
L7 (species)	13.319	0.25953	0.001*	21.899	0.3656	0.001*	10.23	0.21211	0.001*

^*^Statistically significant value.

**Fig. 5:**
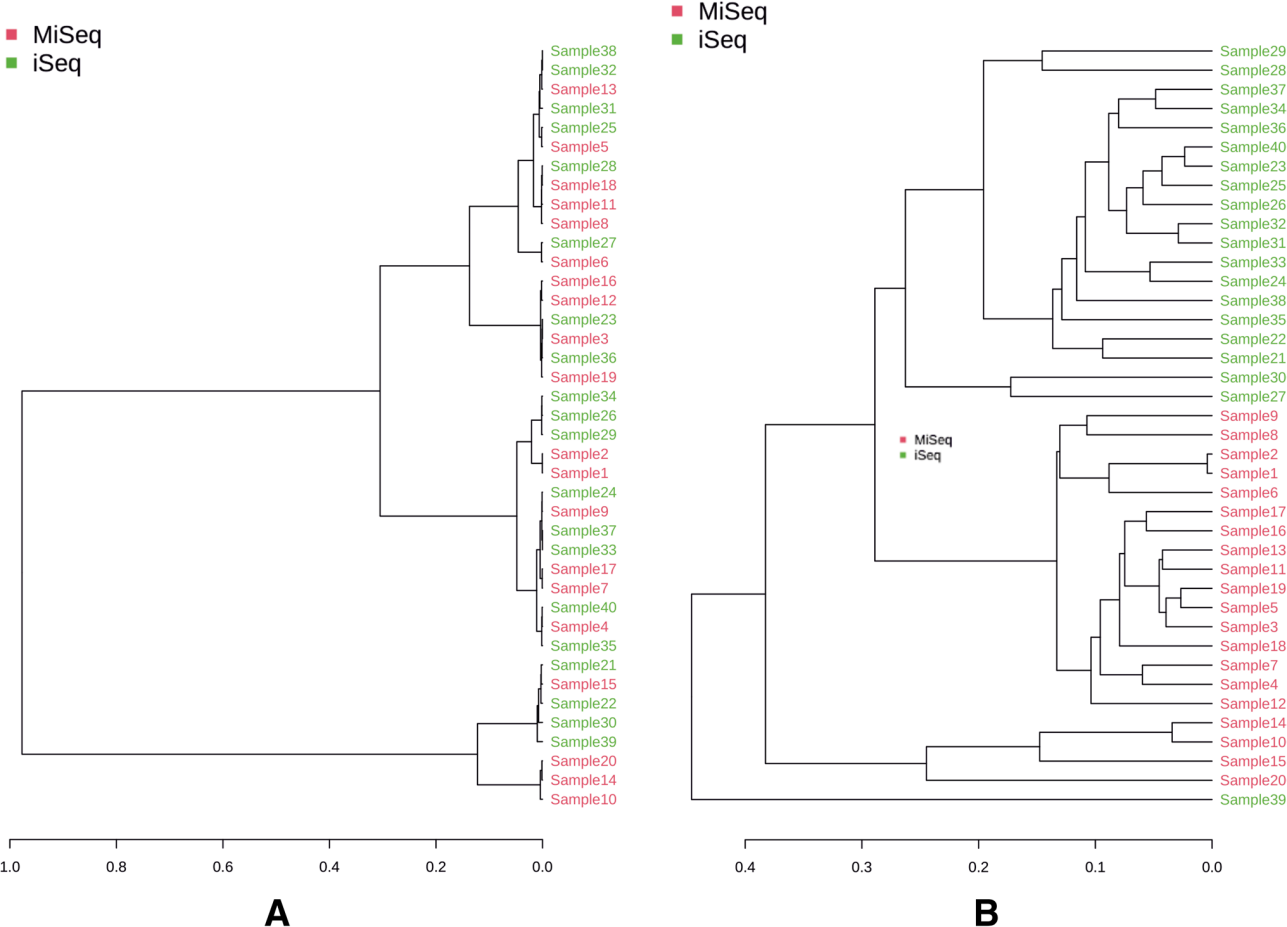
The dendrograms demonstrate the concordance in beta diversity MiSeq and iSeq at phylum levels (**A**) and species levels (**B**). The x-axis shows the cluster distances. The x-axis shows the cluster distances. The distance between two clusters is the average of the distances between all the features in those clusters

A phylogenetic comparison, using unweighted UniFrac distance measures, of the samples sequenced on both tested sequencers was also performed and demonstrated the differences (Fig. 5). A dendrogram was represented by a row graph showing the clades (as the branches) with the leaves. This analysis revealed the different distances between samples in the clustering input for the results obtained in two different sequencers. In addition, the clades show similarities and dissimilarities. For example, clades close to the same height are similar, and clades with different sizes are dissimilar — the more significant the height difference, the more dissimilarity is present.

## Discussion

This study compared two short-read Illumina platforms: iSeq 100 and MiSeq systems. They enable the second-generation sequencing, relating to the NGS technologies after the first-generation Sanger sequencing (Das et al. 2020; Hu et al. 2021). Illumina’s sequencing systems are currently most popular used in microbiome studies (Gao et al. 2021; Nakao et al. 2021).

Due to the same techniques of genomic library preparation and bioinformatics evaluation of sequenced 16S RNA gene fragments, both platforms seem helpful to the same extent. However, if we consider the equipment’s size, cost and the sequencing’s time duration (Table 1), iSeq seems to be the platform of choice (Colman et al. 2019; Dohál et al. 2020; Kazantseva et al. 2021). Additionally, a close model of the cartridge-made iSeq platform may have a lower cross-contamination risk between sequencing runs than in MiSeq (Nakao et al. 2021), although future research should evaluate cross-contamination risks as additional system’s maintenance procedures were introduced.

Nakao et al. (2021) considered that both platforms were equally suitable for evaluating freshwater fish environmental microbiome, and the % PF value for iSeq was slightly lower compared to MiSeq: 80.8% vs. 95.05%, respectively. Uelze L. et al. compared the consistency, accuracy, and repeatability of whole-genome short read sequencing. They found that the four Illumina platforms (MiSeq, NextSeq, iSeq, NovaSeq) gave similar results with slight variation among the different Illumina instruments. However the researchers looked for a few selected bacterial strains Uelze et al. (2020). Our comparative analysis shows a more significant difference in the % PF (passing filter) value (74% for iSeq vs 92% for MiSeq—Table 1), which was more concordant with the data provided by (Brun et al. (2018) (67.6% for iSeq vs 74.9% for MiSeq). In our study, we used an increased concentration of PhiX at the level of 30%, which allowed us to optimize the sequencing process and which has already been used in our other study (Kowalska-Duplaga et al. 2019). Additionally, our study found a higher number of identified bacterial species when MiSeq was used rather than iSeq (9184 vs 7026 respectively), which may be necessary in the final evaluation of the investigated microbiomes. We showed the scale of this difference by assessing the technical aspects of iSeq and MiSeq, comparing the sequencing results on two platforms of one randomly selected sample (Fig. 2).

The results of Nakao et al. (2021) indicate that the freshwater fish microbiota exhibited differences in the number of species between iSeq and MiSeq — a higher number of species per sample in iSeq. However, for example, at L4, all species detected only by iSeq, after rarefaction, were below the cut-off value. The authors suggested that for the same sequence depth, the difference in the number of species between iSeq and MiSeq will be much smaller (Nakao et al. 2021). Our rarefaction curve analysis demonstrated that the species richness of iSeq technology was statistically lower than that of MiSeq (Fig. 1). That meant that most of the OTUs detected with iSeq were the same species, unlike MiSeq, where more species could be identified. This fact seemed to be confirmed by the abundance results at the different taxonomic levels (Fig. 2 and Supplemental Table S2). Furthermore, the discrepancies with a different substantial proportion of taxa at the species level (Fig. 4) suggested that the MiSeq platform would be more beneficial for detecting less known bacteria.

The results comparing alpha and beta diversity (Finotello et al. 2018) were also significantly different for both platforms, starting at the order level, and were most pronounced at the species level. Lower alpha diversity in the samples sequenced on the iSeq platform indicated less biodiversity and, therefore smaller number of species in a single sample (minor compositional complexity of a community within the site). In turn, the differences in beta diversity allowed us to assume that the identified taxa obtained from the iSeq platform were completely different from those sequenced on the MiSeq platform. It would be particularly evident at the species level, as shown in the dendrogram (Fig. 5b). Our comparative studies suggest that choosing a sequencer to evaluate the microbiome is essential and depends on various factors. When analyzing the technical aspect of a randomly selected sample, we noticed the differences in the obtained results due to the platform used. Received results indicated significant differences in relative abundance at the phylum and family levels (Fig. 2). In such a situation, it is impossible to assume that the two sequencers can be used interchangeably to evaluate the gut microbiome.

The mentioned observations suggest that selecting a specific sequencer to assess the microbiome is more critical than the authors postulate, who found no significant difference in the results obtained from iSeq and MiSeq (Uelze et al. 2020; Nakao et al. 2021).Moreover, the limitations of the iSeq system were highlighted by Román-Reyna et al. (2021). They used metagenomics to identify plant pathogenic bacterium *Xylella fastidiosa* down to the strain level in various plant samples. They could not recover the complete sequences of the seven genes of this bacterium and the sequence types were not determined for any field sample. The authors believed this was probably associated with low genome coverage during sequencing by this sequencer (Román-Reyna et al. 2021). It may be necessary for the interpretetion the other studies results where sequencing was carried out on different platforms and the data obtained were evaluated in aggregate without taking into account the type of sequencer (e.g., virological studies on COVID-19) (Lu et al. 2020; Bhoyar et al. 2021). The above data disclose that the iSeq 100 system may be used to evaluate the bacterial profile of the samples to create an overall picture. However, because of twice the read lengths (MiSeq – 2 × 300 base pairs vs iSeq – 2 × 150 bp) and more than six times the maximum reads per run (MiSeq – 25 million sequencing reads vs iSeq – 4 million sequencing reads) the MiSeq system allows for greater probability of a correct, more precise identification of microorganisms at the levels of genus and species, therefore it seems to be better for a detailed analysis of the differences in the microbiota composition of the studied samples.

The weakness of this analysis was a small number of samples and the fact that they come from only animals, which made it difficult to interpret the specific results for all bacterial taxa. However, this work aimed to assess the technical characteristics of the two sequencers and not to solve the investigation results of the particular microbiome. In addition, it was one of the first studies to evaluate the efficiency of the two recommended NGS sequencing platforms on the example of the gut microbiome. https://ruj.uj.edu.pl/xmlui/handle/item/298521.

## Supplementary information

Below is the link to the electronic supplementary material.

Supplementary file1 (PDF 1067 KB)

## Data Availability

Detailed research data is available in the supplementation materials. Fastq files are available The Jagiellonian University Repository—online access:

## References

[CR1] Allali I, Arnold JW, Roach J, Cadenas MB, Butz N, Hassan HM, Koci M, Ballou A, Mendoza M, Ali R, Azcarate-Peril MA (2017). A comparison of sequencing platforms and bioinformatics pipelines for compositional analysis of the gut microbiome. BMC Microbiol.

[CR2] Bellec L, Cambon-Bonavita MA, Durand L, Aube J, Gayet N, Sandulli R, Brandily C, Zeppilli D (2020). Microbial communities of the shallow-water hydrothermal vent near Naples, Italy, and chemosynthetic symbionts associated with a free-living marine nematode. Front Microbiol.

[CR3] Bhoyar RC, Senthivel V, Jolly B, Imran M, Jain A, Divakar MK, Scaria V, Sivasubbu S (2021). An optimized, amplicon-based approach for sequencing of SARS-CoV-2 from patient samples using COVIDSeq assay on Illumina MiSeq sequencing platforms. STAR Protoc.

[CR4] Brun M, Stull MA, Howard E, Hill J, Metz R, Johnson CD (2018) Comparison of MiSeq, iSeq and NovaSeq. In: Texas A&M AgriLife Genomics Bioinforma Serv, pp 2–4. https://www.txgen.tamu.edu/wp-content/uploads/2018/09/iSeq_MiSeq_NovaSeq-test_v03-1.pdf

[CR5] Cabello-Yeves PJ, Callieri C, Picazo A, Mehrshad M, Haro-Moreno JM, Roda-Garcia JJ, Dzhembekova N, Slabakova V, Slabakova N, Moncheva S, Rodriguez-Valera F (2021). The microbiome of the Black Sea water column analyzed by shotgun and genome centric metagenomics. Environ Microbiomes.

[CR6] Chong J, Liu P, Zhou G, Xia J (2020). Using MicrobiomeAnalyst for comprehensive statistical, functional, and meta-analysis of microbiome data. Nat Protoc.

[CR7] Colman RE, Mace A, Seifert M, Hetzel J, Mshaiel H, Suresh A, Lemmer D, Engelthaler DM, Catanzaro DG, Young AG, Denkinger CM, Rodwell TC (2019). Whole-genome and targeted sequencing of drug-resistant *Mycobacterium tuberculosis* on the iSeq100 and MiSeq: A performance, ease-of-use, and cost evaluation. PLoS Med.

[CR8] Das P, Dawal R, Radhakrishnan V, Parihar M, Bhattacharya S, Mishra DK, Chandy M (2020). Comparison of four high throughput sequencing platforms in a medical laboratory for gut microbiome research. Indian J Anim Heal.

[CR9] Dohál M, Porvazník I, Pršo K, Rasmussen EM, Solovič I, Mokrý J (2020). Whole-genome sequencing and *Mycobacterium tuberculosis*: challenges in sample preparation and sequencing data analysis. Tuberculosis.

[CR10] Finotello F, Mastrorilli E, Di Camillo B (2018). Measuring the diversity of the human microbiota with targeted next-generation sequencing. Brief Bioinform.

[CR11] Foster ZSL, Sharpton TJ, Grünwald NJ (2017). Metacoder: An R package for visualization and manipulation of community taxonomic diversity data. PLoS Comput Biol.

[CR12] Gao B, Chi L, Zhu Y, Shi X, Tu P, Li B, Yin J, Gao N, Shen W, Schnabl B (2021). An introduction to next generation sequencing bioinformatic analysis in gut microbiome studies. Biomolecules.

[CR13] Gosiewski T, Szała L, Pietrzyk A, Brzychczy-Włoch M, Heczko PB, Bulanda M (2014). Comparison of methods for isolation of bacterial and fungal DNA from human blood. Curr Microbiol.

[CR14] Hu T, Chitnis N, Monos D, Dinh A (2021). Next-generation sequencing technologies: An overview. Hum Immunol.

[CR15] Kazantseva J, Malv E, Kaleda A, Kallastu A, Meikas A (2021). Optimisation of sample storage and DNA extraction for human gut microbiota studies​. BMC Microbiol.

[CR16] Klindworth A, Pruesse E, Schweer T, Peplies J, Quast C, Horn M, Glöckner FO (2013). Evaluation of general 16S ribosomal RNA gene PCR primers for classical and next-generation sequencing-based diversity studies. Nucleic Acids Res.

[CR17] Kowalska-Duplaga K, Gosiewski T, Kapusta P, Sroka-Oleksiak A, Wędrychowicz A, Pieczarkowski S, Ludwig-Słomczyńska AH, Wołkow PP, Fyderek K (2019). Differences in the intestinal microbiome of healthy children and patients with newly diagnosed Crohn’s disease. Sci Rep.

[CR18] Krawczyk A, Salamon D, Kowalska-Duplaga K, Bogiel T, Gosiewski T (2021). Association of fungi and archaea of the gut microbiota with Crohn’s disease in pediatric patients—pilot study. Pathogens.

[CR19] Lau JT, Whelan FJ, Herath I, Lee CH, Collins SM, Bercik P, Surette MG (2016). Capturing the diversity of the human gut microbiota through culture-enriched molecular profiling. Genome Med.

[CR20] Lu R, Zhao X, Li J, Niu P, Yang B, Wu H, Wang W, Song H, Huang B, Zhu N, Bi Y, Ma X, Zhan F, Wang L, Hu T, Zhou H, Hu Z, Zhou W, Zhao L, Chen J, Meng Y, Wang J, Lin Y, Yuan J, Xie Z, Ma J, Liu WJ, Wang D, Xu W, Holmes EC, Gao GF, Wu G, Chen W, Shi W, Tan W (2020). Genomic characterisation and epidemiology of 2019 novel coronavirus: implications for virus origins and receptor binding. Lancet.

[CR21] Nakao R, Inui R, Akamatsu Y, Goto M, Doi H, Matsuoka S (2021). Illumina iSeq 100 and MiSeq exhibit similar performance in freshwater fish environmental DNA metabarcoding. Sci Rep.

[CR22] Román-Reyna V, Dupas E, Cesbron S, Marchi G, Campigli S, Hansen MA, Bush E, Prarat M, Shiplett K, Ivey MLL, Pierzynski J, Miller SA, Peduto Hand F, Jacques M-A, Jacobs JM (2021) Metagenomic sequencing for identification of *Xylella fastidiosa* from leaf samples. mSystems 6(5):e00591-21. 10.1128/MSYSTEMS.00591-21 10.1128/mSystems.00591-21 PMC8547472 34698548

[CR23] Salamon D, Sroka-Oleksiak A, Kapusta P, Szopa M, Mrozińska S, Ludwig-Słomczyńska AH, Wołkow PP, Bulanda M, Klupa T, Małecki MT, Gosiewski T (2018). Characteristics of gut microbiota in adult patients with type 1 and type 2 diabetes based on next-generation sequencing of the 16S rRNA gene fragment. Polish Arch Intern Med.

[CR24] Salamon D, Sroka-Oleksiak A, Gurgul A, Arent Z, Szopa M, Bulanda M, Małecki MT, Gosiewski T (2021). Analysis of the gut mycobiome in adult patients with type 1 and type 2 diabetes using next-generation sequencing (NGS) with increased sensitivity—pilot study. Nutrients.

[CR25] Schloss PD, Westcott SL, Ryabin T, Hall JR, Hartmann M, Hollister EB, Lesniewski RA, Oakley BB, Parks DH, Robinson CJ, Sahl JW, Stres B, Thallinger GG, Van Horn DJ, Weber CF (2009). Introducing mothur: Open-source, platform-independent, community-supported software for describing and comparing microbial communities. Appl Environ Microbiol.

[CR26] Segata N, Izard J, Waldron L, Gevers D, Miropolsky L, Garrett WS, Huttenhower C (2011). Segata-LEfSe-gb-2011. Genome Biol.

[CR27] Sroka-Oleksiak A, Młodzińska A, Bulanda M, Salamon D, Major P, Stanek M, Gosiewski T (2020). Metagenomic analysis of duodenal microbiota reveals a potential biomarker of dysbiosis in the course of obesity and type 2 diabetes: a pilot study. J Clin Med.

[CR28] Szeligowski T, Yun AL, Lennox BR, Burnet PWJ (2020). The gut microbiome and schizophrenia: the current state of the field and clinical applications. Front Psychiatry.

[CR29] Uelze L, Borowiak M, Bönn M, Brinks E, Deneke C, Hankeln T, Kleta S, Murr L, Stingl K, Szabo K, Tausch SH, Wöhlke A, Malorny B (2020). German-wide interlaboratory study compares consistency, accuracy and reproducibility of whole-genome short read sequencing. Front Microbiol.

[CR30] Vasco K, Nohomovich B, Singh P, Venegas-Vargas C, Mosci RE, Rust S, Bartlett P, Norby B, Grooms D, Zhang L, Manning SD (2021). Characterizing the cattle gut microbiome in farms with a high and low prevalence of shiga toxin-producing *Escherichia coli*. Microorganisms.

[CR31] Wang Q, Garrity GM, Tiedje JM, Cole JR (2007). Naïve Bayesian classifier for rapid assignment of rRNA sequences into the new bacterial taxonomy. Appl Environ Microbiol.

[CR32] Zhang J, Cook J, Nearing JT, Zhang J, Raudonis R, Glick BR, Langille MGI, Cheng Z (2021). Harnessing the plant microbiome to promote the growth of agricultural crops. Microbiol Res.

