# Supplementary material for: Comparison of iSeq and MiSeq as the two platforms for 16S rRNA sequencing in the study of the gut of rat microbiome
Source: Appl Microbiol Biotechnol. 2022 Nov 2;106(22):7671–81. doi: 10.1007/s00253-022-12251-z (PMC9628524; doi:10.1007/s00253-022-12251-z)
Supplement: Supplementary file 1 — Supplementary file1 (PDF 1067 KB) [file 253_2022_12251_MOESM1_ESM.pdf]

Applied Microbiology and Biotechnology

Supporting information for:

Comparison of iSeq and MiSeq as the two platforms for 16S rRNA sequencing in the study of the gut of rats microbiome

Dominika Salamon[1#], Barbara Zapała[2#], Agnieszka Krawczyk[1], Agnieszka Potasiewicz[3], Agnieszka Nikiforuk[3], Anastazja Stój[4], Tomasz Gosiewski[1*]

[1] Jagiellonian University Medical College, Faculty of Medicine, Chair of Microbiology, Department of Molecular Medical Microbiology, Krakow, Poland.

[2] Jagiellonian University Medical College, Faculty of Medicine, Department of Clinical Biochemistry, Krakow, Poland

[3] Maj Institute of Pharmacology, Polish Academy of Sciences, Department of Behavioural Neuroscience and Drug Development, Krakow, Poland

[4] Department of Hematology Diagnostics, The University Hospital, 30-688 Cracow, Poland

* Corresponding author: tomasz.gosiewski@uj.edu.pl (TG) , Tel.: +4812-633-25-67

# D.S. and B.Z. contributed equally to this work as first authors.

<u>This document contains:</u>

Tables S1 to S4

Figures S1 to S11

TABLES

Table S1: Primer sequences (Genomed, Warsaw, Poland), reaction mixtures, and thermal amplification profiles were used in the study.

Primer sequence 5'→3'	Reaction mixture	Thermal amplification profile
<u>Primers</u>[a]		
F: TCGTCGGCAGCGTCAGATGTGTATAAGAGACAGCCTACGGGNGGCWGCAG<br>R: GTCTCGTGGGCTCGGAGATGTGTATAAGAGACAGGACTACHVGGGTATCTAATCC	Molecular grade water[b] - 10.5 µl<br>Kapa[c] - 12.5 µl<br>Pr. F (10 µM) - 0.5 µl<br>Pr. R (10 µM) - 0.5 µl<br>DNA - 1.0µl	95 °C – 3 min<br>95 °C – 30 sec } 30x<br>55 °C – 30 sec } 30x<br>72 °C – 30 sec } 30x<br>72 °C – 5 min

[a] reference (Klindworth et al. 2013)

[b] Molecular grade water (Roche Kapa Biosystems, Wilmington, United States)

[c] 2x KAPA HiFi HotStart ReadyMix (Roche Kapa Biosystems, Wilmington, United States)

Table S2: The statistically significant differences in the OTUs abundance at three taxonomic levels (family-L5, genus-L6, and species-L7).

**FAMILY (L5)**			
**Taxonomy level**	**iSeq (number of classified reads)**	**MiSeq (number of classified reads)**	***p*-value**
Bacteroidaceae	1228	77	**0.023**
Barnesiellaceae	1636	7138	**0.002**
Bifidobacteriaceae	80542	44994	**0.042**
Erysipelatoclostridiaceae	1180	33	**0.000**
Erysipelotrichaceae	572102	326277	**0.003**
Nocardioidaceae	1848	157	**0.001**
Peptostreptococcaceae	2893	282221	**0.000**
Promicromonosporaceae	2	97253	**0.049**
Tannerellaceae	1903	184	**0.009**
Thermoactinomycetaceae	2637	144	**0.000**
**GENUS (L6)**			
**Taxonomy level**	**iSeq**	**MiSeq**	***p*-value**
Acutalibacter	5165	961	**0.003**
Adlercreutzia	19961	4738	**0.013**
Acutalibacter	5165	961	**0.003**
Adlercreutzia	19961	4738	**0.013**
Bacteroides	1228	77	**0.023**
Barnesiella	1636	7138	**0.002**
Bifidobacterium	80542	44994	**0.042**
Blautia	2508	13120	**0.048**
Cellulosimicrobium	2	97253	**0.049**
Eggerthella	30783	4273	**0.002**
Erysipelatoclostridium	1180	33	**0.003**
Faecalibaculum	118755	44268	**0.029**
Intestinimonas	4561	2466	**0.041**
Micropruina	1848	157	**0.001**
Parabacteroides	1903	184	**0.009**

Paraclostridium	1426	52	**0.000**
Paraeggerthella	177	9545	**0.035**
Pseudoflavonifractor	2961	1345	**0.036**
Romboutsia	1467	282169	**0.000**
Salinithrix	2637	144	**0.000**
Slackia	655	7673	**0.004**
Turicibacter	444072	267614	**0.007**
**SPECIES (L7)**			
**Taxonomy level**	**iSeq**	**MiSeq**	***p*-value**
Lactobacillus gasseri	0	5386600	**0.000**
Cellulosimicrobium funkei	30965	9365000	**0.000**
Streptococcus peroris	41911	229090	**0.000**
Romboutsia ilealis	75832	865970	**0.000**
Hydrogenibacillus schlegelii	0	259910	**0.000**
Clostridium hylemonae	14854	690200	**0.000**
Rothia nasimurium	19677	1457000	**0.000**
Romboutsia timonensis	66323	24694000	**0.000**
Clostridium innocuum	120390	23066	**0.000**
Lactobacillus johnsonii	8407900	496650	**0.000**
Paraclostridium bifermentans	140340	42066	**0.000**
Hungateiclostridium cellulolyticum	29631	140740	**0.000**
Barnesiella viscericola	63275	501300	**0.000**
Paraeggerthella hongkongensis	16673	614470	**0.000**
Thermoactinomycetaceae bacterium	236250	12137	**0.000**
Rothia dentocariosa	1941300	21264	**0.000**
Micropruina glycogenica	183750	20175	**0.000**
Clostridium hiranonis	132040	21916	**0.000**
Eggerthella sinensis	2930100	412140	**0.000**
Slackia equolifaciens	63522	742730	**0.000**

Streptococcus acidominimus	51355	317080	**0.000**
Blautia producta	132440	12680	**0.000**
Clostridium quinii	185810	22215	**0.000**
Acutalibacter muris	449180	114830	**0.002**
Dorea longicatena	298010	43855	**0.002**
Christensenella timonensis	214840	45360	**0.003**
Adlercreutzia equolifaciens	1827500	501180	**0.005**
Turicibacter sanguinis	43753000	27906000	**0.005**
Parabacteroides chartae	193540	27058	**0.006**
Faecalibaculum rodentium	11057000	4711000	**0.013**
Clostridium lavalense	106170	16106	**0.016**
Barnesiella intestinihominis	150120	332160	**0.016**
Clostridium sp	83249	56898	**0.021**
Bacteroides mediterraneensis	120620	87508	**0.025**
Acetivibrio ethanolgignens	30285	79817	**0.033**
Blautia glucerasea	144930	1428400	**0.040**
Flintibacter butyricus	425890	198340	**0.040**
Bifidobacterium animalis	7340000	4356800	**0.045**

Table S3: The features with significant differential abundance at the genus level. The statistically significant ($p < 0.05$) features are ranked by their *p* values, in the same samples sequenced in MiSeq and iSeq machines respectively.

**Genus**	**p-value**	**MiSeq**	**iSeq**
Cellulosimicrobium	0.000	936500	30
Hydrogenibacillus	0.000	25991	0
Romboutsia	0.000	2561700	15033
Erysipelatoclostridium	0.000	230	12039
Paraclostridium	0.000	420	14034
Paraeggerthella	0.000	61447	1667
Salinithrix	0.000	1213	23625
Micropruina	0.000	2017	18375
Eggerthella	0.000	41214	293010
Slackia	0.000	74273	6352
Barnesiella	0.000	83346	15645
Acutalibacter	0.001	11483	44918
Dorea	0.001	4385	29801
Christensenella	0.002	4536	21484
Adlercreutzia	0.004	50118	182750
Turicibacter	0.005	2790600	4375300
Parabacteroides	0.005	2705	19354
Faecalibaculum	0.012	471100.0	1105700
Bacteroides	0.024	875	12062
Acetivibrio	0.032	7981	3028
Intestinimonas	0.039	19834	42589
Bifidobacterium	0.045	435680	734000

Table S4: The features with significant differential abundance at the species level. The statistically significant ($p < 0.05$) features are ranked by their *p* values, in the same samples sequenced in MiSeq and iSeg machines respectively.

**Species**	**p-value**	**MiSeq**	**iSeq**
Lactobacillus gasseri	0.000	538660	0
Cellulosimicrobium funkei	0.000	936500	31
Streptococcus peroris	0.000	22909	42
Romboutsia ilealis	0.000	86597	76
Hydrogenibacillus schlegelii	0.000	25991	0
Clostridium hylemonae	0.000	69020	15
Rothia nasimurium	0.000	145700	197
Romboutsia timonensis	0.000	2469400	6632
Clostridium innocuum	0.000	231	12039
Lactobacillus johnsonii	0.000	49665	840790
Paraclostridium bifermentans	0.000	421	14034
Hungateiclostridium cellulolyticum	0.000	14074	30
Barnesiella viscericola	0.000	50130	633
Paraeggerthella hongkongensis	0.000	61447	1667
Thermoactinomycetaceae bacterium	0.000	1214	23625
Rothia dentocariosa	0.000	213	194130
Micropruina glycogenica	0.000	2018	18375
Clostridium hiranonis	0.000	2192	13204
Eggerthella sinensis	0.000	41214	293010
Slackia equolifaciens	0.000	74273	6352
Streptococcus acidominimus	0.000	31708	5136
Blautia producta	0.000	1268	13244
Clostridium quinii	0.000	2222	18581
Acutalibacter muris	0.001	11483	44918
Dorea longicatena	0.001	4386	29801
Christensenella timonensis	0.002	4536	21484

Adlercreutzia equolifaciens	0.004	50118	182750
Turicibacter sanguinis	0.005	2790600	4375300
Parabacteroidechartae	0.005	2706	19354
Faecalibaculum rodentium	0.012	471100	1105700
Clostridium lavalense	0.015	1611	10617
Barnesiella intestinihominis	0.016	33216	15012
Clostridium sp	0.021	5690	8325
Bacteroidemediterraneensis	0.024	875	12062
Acetivibrio ethanolgignens	0.032	7982	3029
Blautia glucerasea	0.039	142840	14493
Flintibacter butyricus	0.039	19834	42589
Bifidobacterium animalis	0.045	435680	734000

FIGURES

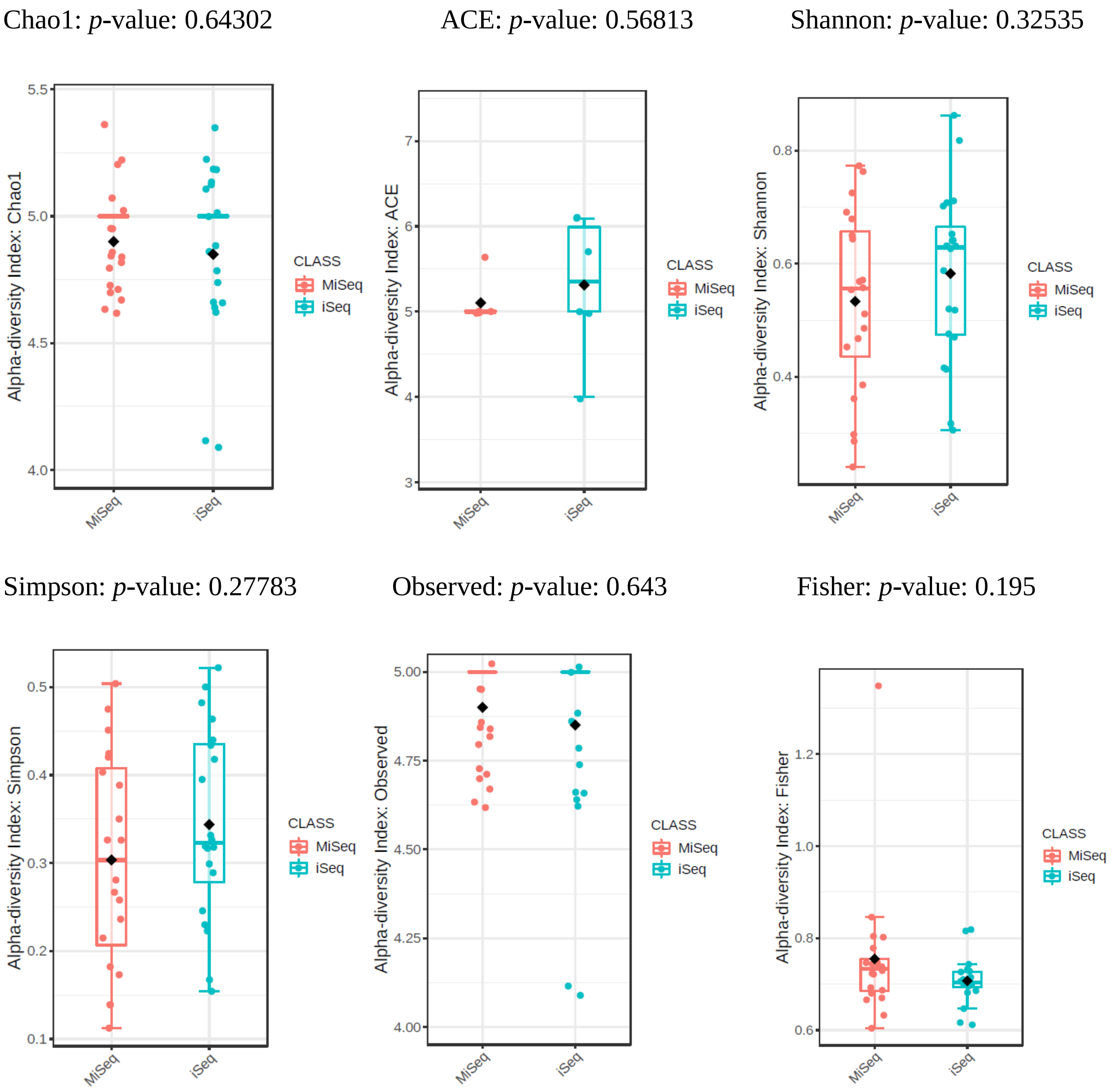

Figure S1: Alfa diversity expressed in six indices at the phylum and class levels.

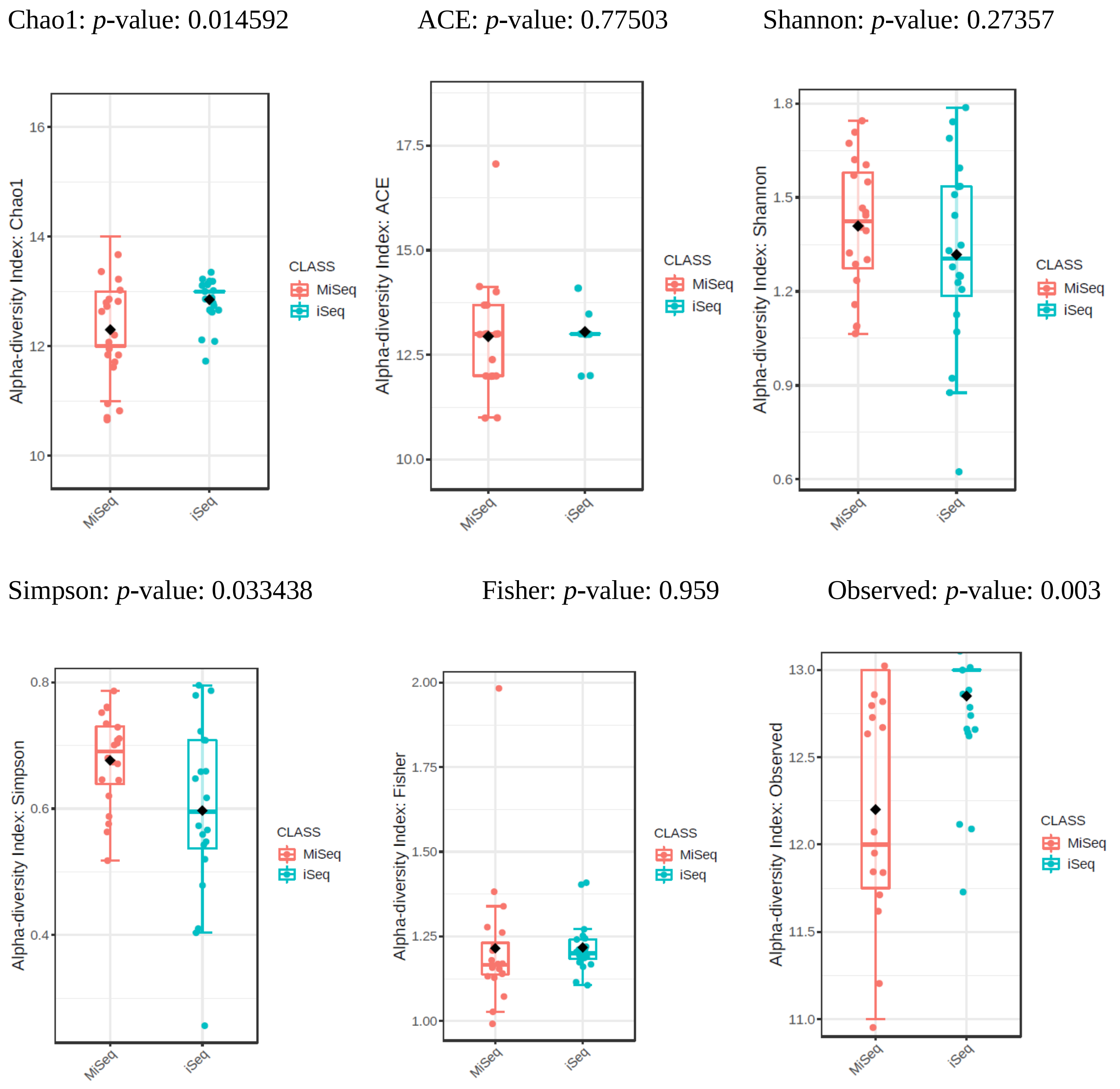

Figure S2: Alfa diversity expressed in six indices at the order level.

Chao1: *p*-value: 0.051934

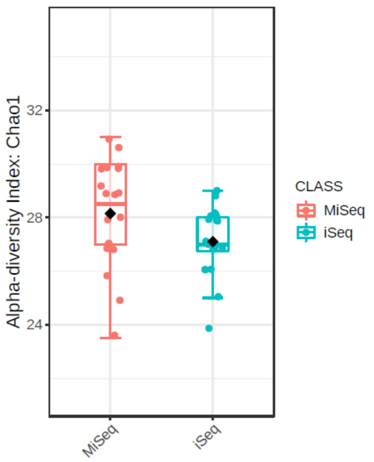

ACE: *p*-value: 0.077

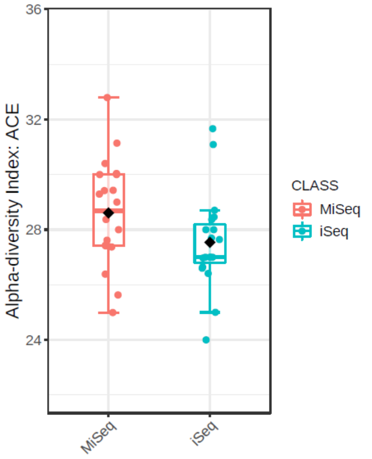

Shannon: *p*-value: 0.052

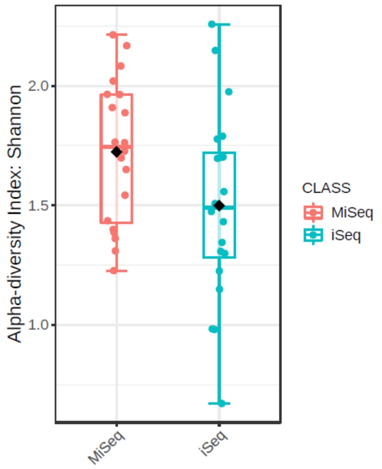

Simpson: *p*-value: 0.004

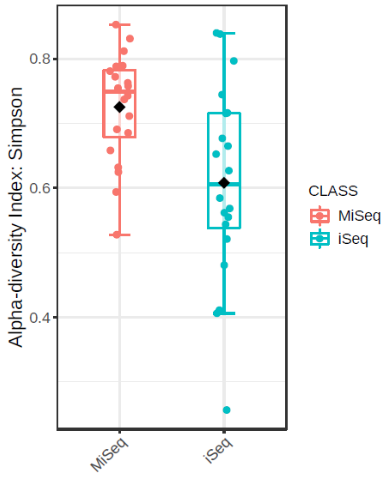

Observed: *p*-value: 0.032

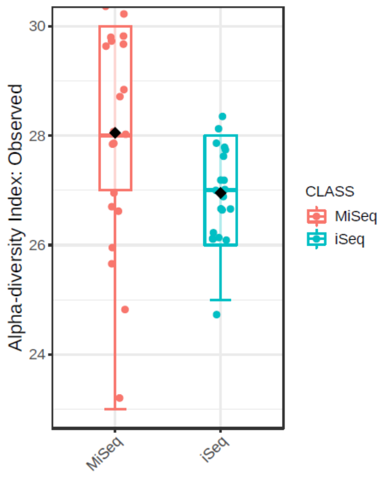

Fisher: *p*-value: 0.015

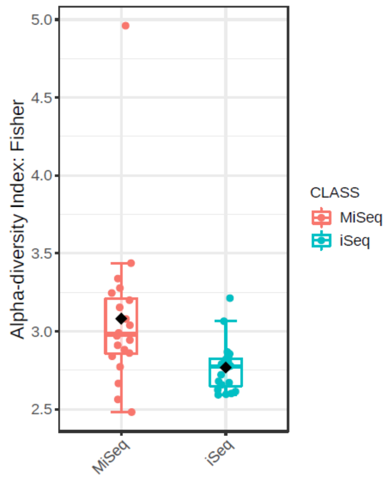

Figure S3: Alfa diversity expressed in six indices at the family level.

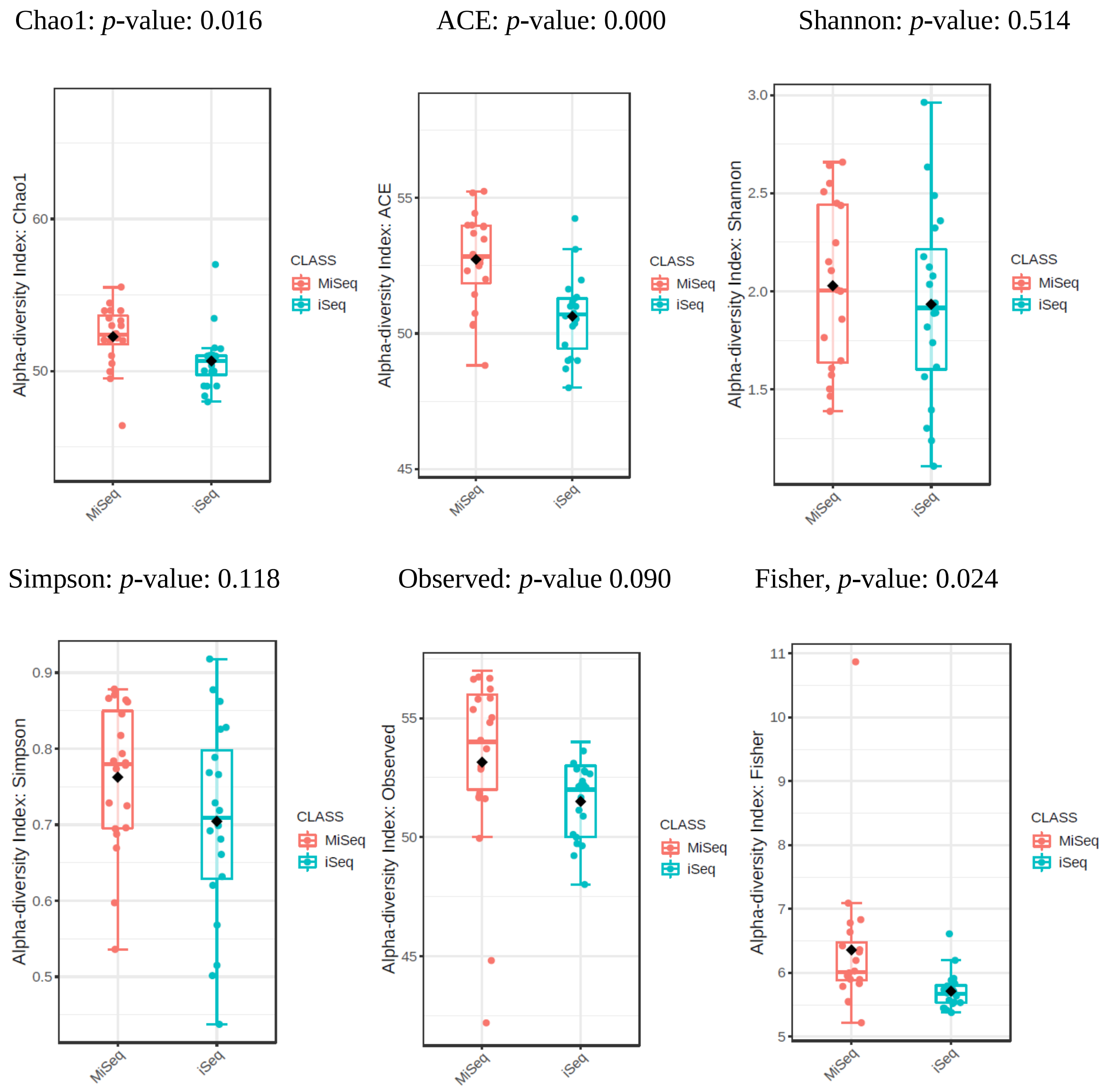

Figure S4: Alfa diversity expressed in six indices at the genus level.

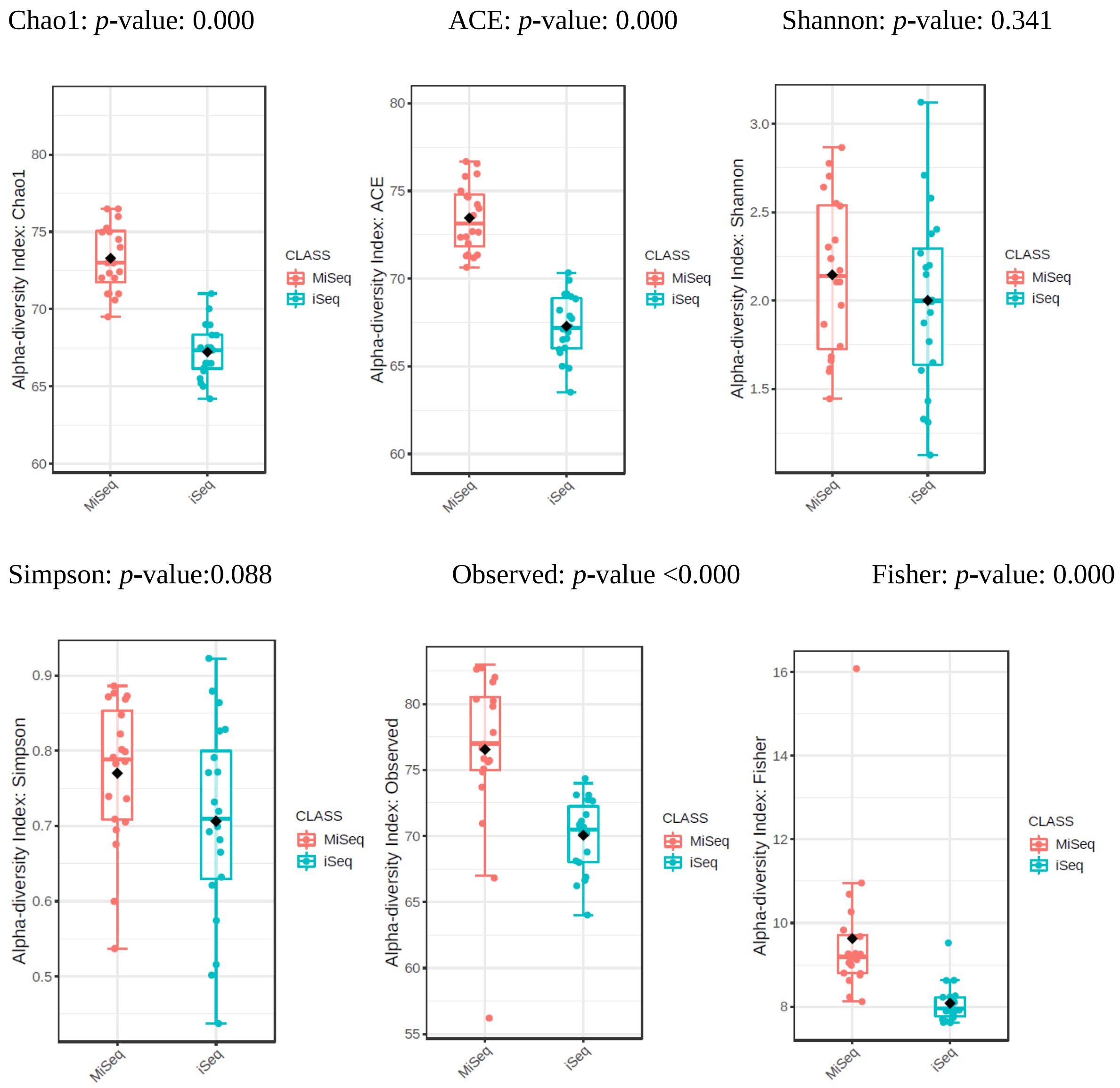

Figure S5: Alfa diversity expressed in six indices at the species level.

[PERMANOVA] F-value: 0.11248; R-squared: 0.0029513; *p*-value < 0.82

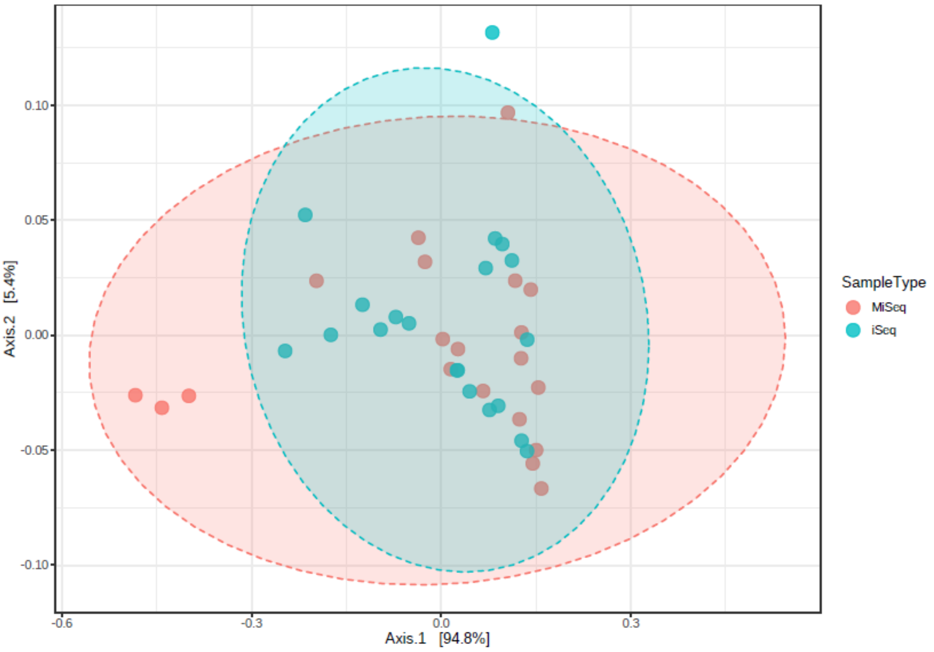

Figure S6: Beta diversity expressed with the Bray-Curtis index at the phylum level, visualized by principal coordinate analysis (PCoA).

[PERMANOVA] F-value: 15.589; R-squared: 0.2909; *p*-value < 0.001

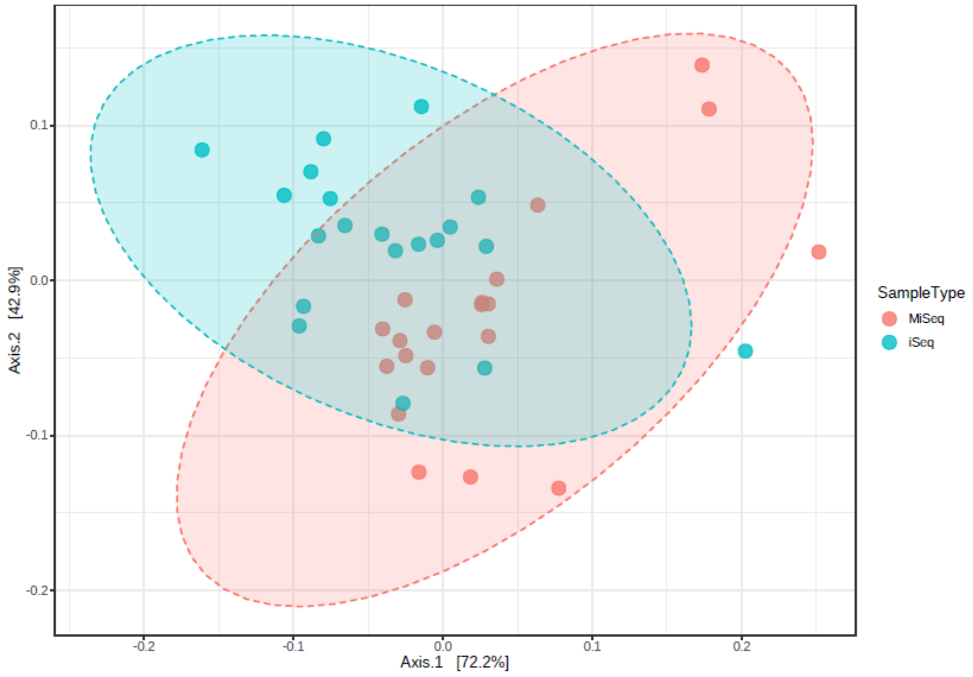

Figure S7: Beta diversity expressed with the Bray-Curtis index at the class level, visualized by principal coordinate analysis (PCoA).

[PERMANOVA] F-value: 16.27; R-squared: 0.29979; *p*-value < 0.001

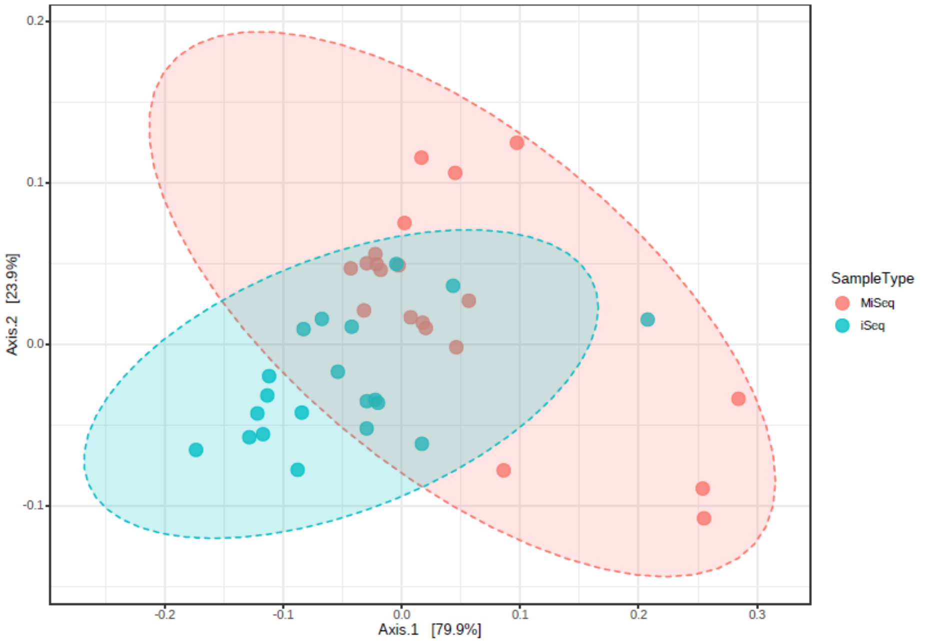

Figure S8: Beta diversity expressed with the Bray-Curtis index at the order level, visualized by principal coordinate analysis (PCoA). A significant correspondence between beta-diversity estimates (MiSeq - red separate from iSeq – blue) were demonstrated.

[PERMANOVA] F-value: 24.182; R-squared: 0.38889; *p*-value < 0.001

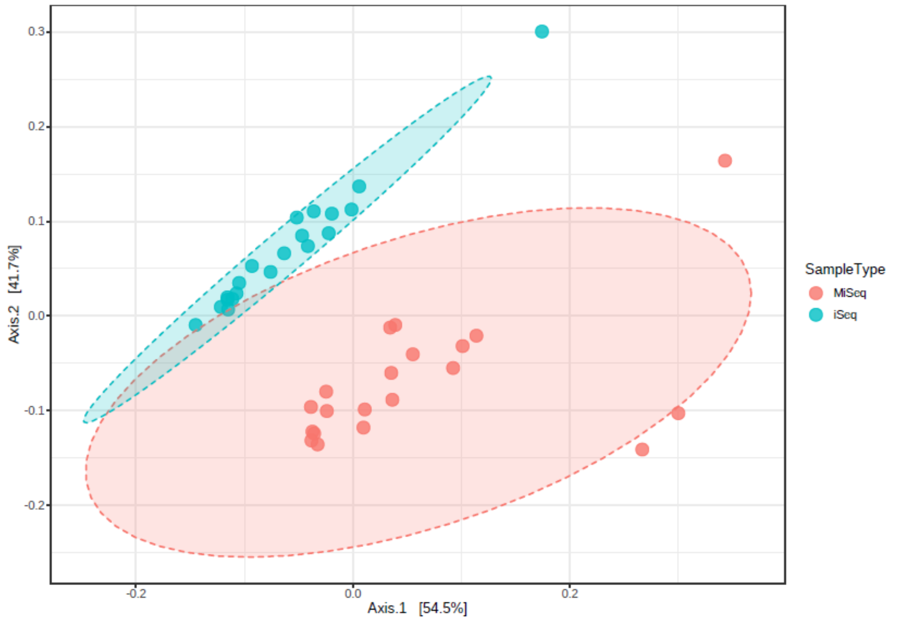

Figure S9: Beta diversity expressed with the Bray-Curtis index at the family level, visualized by principal coordinate analysis (PCoA). A significant correspondence between beta-diversity estimates (MiSeq - red separate from iSeq – blue) were demonstrated.

[PERMANOVA] F-value: 21.899; R-squared: 0.3656; *p*-value < 0.001

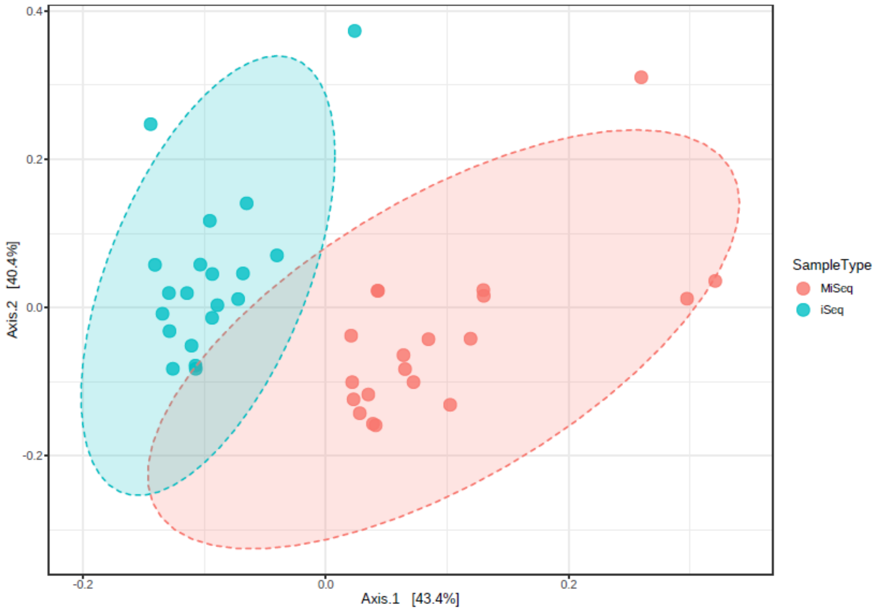

Figure S10: Beta diversity expressed with the Bray-Curtis index at the genus level, visualized by principal coordinate analysis (PCoA). A significant correspondence between beta-diversity estimates (MiSeq - red separate from iSeq – blue) were demonstrated.

[PERMANOVA] F-value: 31.408; R-squared: 0.45251; *p*-value < 0.001

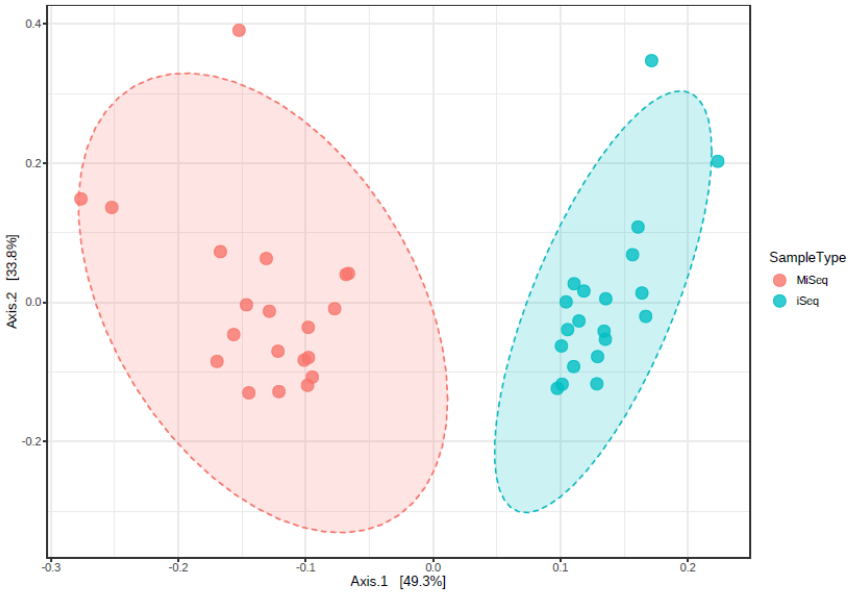

Figure S11: Beta diversity expressed with the Bray-Curtis index at the species level, visualized by principal coordinate analysis (PCoA). A significant correspondence between beta-diversity estimates (MiSeq - red separate from iSeq – blue) were demonstrated.
